# Telocinobufagin, a Novel Cardiotonic Steroid, Promotes Renal Fibrosis via Na^+^/K^+^-ATPase Profibrotic Signaling Pathways

**DOI:** 10.3390/ijms19092566

**Published:** 2018-08-29

**Authors:** David J. Kennedy, Fatimah K. Khalaf, Brendan Sheehy, Malory E. Weber, Brendan Agatisa-Boyle, Julijana Conic, Kayla Hauser, Charles M. Medert, Kristen Westfall, Philip Bucur, Olga V. Fedorova, Alexei Y. Bagrov, W. H. Wilson Tang

**Affiliations:** 1 Department of Medicine, University of Toledo College of Medicine, Toledo, OH 43614, USA; David.Kennedy@UToledo.edu (D.J.K.); kareem.khalaf@rockets.utoledo.edu (F.K.K.); 2 Department of Cellular and Molecular Medicine, Lerner Research Institute Cleveland Clinic, Cleveland, OH 44106, USA; bts21@case.edu (B.S.); weberm9@ccf.org (M.E.W.); bcaboyle@gmail.com (B.A.-B.); julijanaconic@gmail.com (J.C.); kvmislick@gmail.com (K.H.); c.maxmedert@gmail.com (C.M.M.); WESTFAK@ccf.org (K.W.); pb403014@ohio.edu (P.B.); 3 Laboratory of Cardiovascular Science, National Institute on Aging, National Institutes of Health, Baltimore, MD 21224, USA; FedorovO@grc.nia.nih.gov; 4 Sechenov Institute of Evolutionary Physiology and Biochemistry, St. Petersburg 194223, Russia; aybagrov@gmail.com; 5 Center for Cardiovascular Diagnostics and Prevention, Lerner Research Institute Cleveland Clinic, Cleveland, OH 44106, USA; 6 Department of Cardiovascular Medicine, Heart and Vascular Institute, Cleveland Clinic, Cleveland, OH 44195, USA

**Keywords:** telocinobufagin, cardiotonic steroids, Na^+^/K^+^-ATPase, kidney, fibrosis, signaling

## Abstract

Cardiotonic steroids (CTS) are Na^+^/K^+^-ATPase (NKA) ligands that are elevated in volume-expanded states and associated with cardiac and renal dysfunction in both clinical and experimental settings. We test the hypothesis that the CTS telocinobufagin (TCB) promotes renal dysfunction in a process involving signaling through the NKA α-1 in the following studies. First, we infuse TCB (4 weeks at 0.1 µg/g/day) or a vehicle into mice expressing wild-type (WT) NKA α-1, as well as mice with a genetic reduction (~40%) of NKA α-1 (NKA α-1^+/−^). Continuous TCB infusion results in increased proteinuria and cystatin C in WT mice which are significantly attenuated in NKA α-1^+/−^ mice (all *p* < 0.05), despite similar increases in blood pressure. In a series of in vitro experiments, 24-h treatment of HK2 renal proximal tubular cells with TCB results in significant dose-dependent increases in both Collagens 1 and 3 mRNA (2-fold increases at 10 nM, 5-fold increases at 100 nM, *p <* 0.05). Similar effects are seen in primary human renal mesangial cells. TCB treatment (100 nM) of SYF fibroblasts reconstituted with cSrc results in a 1.5-fold increase in Collagens 1 and 3 mRNA (*p <* 0.05), as well as increases in both Transforming Growth factor beta (TGFb, 1.5 fold, *p <* 0.05) and Connective Tissue Growth Factor (CTGF, 2 fold, *p <* 0.05), while these effects are absent in SYF cells without Src kinase. In a patient study of subjects with chronic kidney disease, TCB is elevated compared to healthy volunteers. These studies suggest that the pro-fibrotic effects of TCB in the kidney are mediated though the NKA-Src kinase signaling pathway and may have relevance to volume-overloaded conditions, such as chronic kidney disease where TCB is elevated.

## 1. Introduction

Patients with chronic kidney disease (CKD) often experience progressive renal compromise, which leads to recurrent hospitalizations and clinical deterioration. Renal dysfunction confers one of the most significant independent risk factors for poor outcomes and all-cause mortality [1]. In fact, the glomerular filtration rate (GFR) predicts mortality more strongly than either left ventricular ejection fraction or New York Heart Association (NYHA) functional class, while worsening renal function during hospitalization predicts longer hospital stays as well as increased rehospitalization rates and mortality [2]. As our mechanistic understanding of CKD is lacking, there are limited treatment options beyond contemporary therapies of neurohormonal blockade.

Cardiotonic steroids (CTS) are a class of endogenous hormones that are elevated in volume-expanded states. These hormones confer a natriuretic response by inducing endocytosis of renal proximal tubule cell Na^+^/K^+^-ATPase [3,4]. By this mechanism, sodium excretion is increased, as the Na^+^/K^+^-ATPase is removed from the basolateral membrane, thus reducing the transport of sodium from the proximal tubular lumen to the blood compartment. 

In addition to their well known effects on the ion transporting functions of the Na^+^/K^+^-ATPase, CTS also bind to and initiated signaling through a non-pumping pool of the Na^+^/K^+^-ATPase which reside in caveolae [3,5]. CTS confer a conformational change to the Na^+^/K^+^-ATPase that releases the Src kinase domain, thus activating Src kinase [6,7,8] and multiple downstream targets [7,9,10,11,12,13]. This novel Na^+^/K^+^-ATPase-mediated signaling directs a number of important cellular functions, including cell growth/hypertrophy [14], reactive oxygen species (ROS) production [15], and collagen synthesis [16,17] among others (reviewed in [3,4,18]). CTS are known to activate both Src and Lyn kinase in vitro [6,19] and in vivo [20] as part of the Na^+^/K^+^-ATPase-mediated signaling cascade.

However, chronic elevation of CTS may also exert pro-inflammatory and pro-fibrotic signaling events in cardiac and renal tissue mediated by the Na^+^/K^+^-ATPase [21,22]. Clinical and experimental evidence from our group and others has also demonstrated the pro-oxidant and pro-fibrotic effects of these steroid hormones in both cardiac and renal tissue [16,17,20,23,24,25,26,27]. The role of Na^+^/K^+^-ATPase signaling in this “trade-off” between natriuresis and end organ hypertrophy and fibrosis has been recently reviewed in detail [22]. A central signaling theme in the binding of CTS to the Na^+^/K^+^-ATPase is a conformational change that releases the kinase domain of the sodium pump, thus activating Src kinase [6,7,8]. Activation of Src kinase transactivates the epidermal growth factor receptor, and results in the activation of the Ras/Raf/MEK/ERK1/2 cascade [10]. This novel Na^+^/K^+^-ATPase-mediated signaling directs a number of important physiologic and pathophysiologic cellular functions [14,28,29,30]. This ability of CTS to activate these Na^+^/K^+^-ATPase signaling events make them attractive therapeutic targets for intervention in CKD. 

CTS can be generally divided into subclasses based on the presence of either a five-membered lactone ring (as in cardienolides species, such as ouabain) or a six-membered lactone ring (such as contained in the bufadienolides telocinobufagin (TCB) or marinobufagenin (MBG) (see Supplementary Materials, Supplemental Figure S1). Important functional differences have been noted within and among the classes of CTS. In comparative studies of Na^+^/K^+^-ATPase inhibitory actions, TCB is 30 times more potent than its relative MBG [31,32], and exhibits the most potent activity of all the CTS studied in both canine and feline myocardial Na^+^/K^+^-ATPase [33]. While TCB is well established as a potent pro-apoptotic extract from toad venom [34,35], as well as one of the major components of the traditional Chinese medicinal preparations of Chan Su [36], it is only within the last decade that it has been definitely measured in the plasma of patients with end stage renal disease [37]. Nevertheless, the role of TCB in antecedent stages or renal dysfunction remains unclear. Thus, in the current study, we sought to determine the ability of TCB to activate pro-fibrotic pathways in renal tissue via Na^+^/K^+^-ATPase signaling. We also measured circulating levels TCB within patients with CKD.

## 2. Methods

### 2.1. In Vivo Studies

Littermate-derived wild-type male 129 SvJ/Black Swiss mice (25–30 g) (WT), as well as 129 SvJ/Black Swiss mice heterozygous for the Na^+^/K^+^-ATPase-α-1 (referred to as NKA α-1^+/−^) constituting a genetic reduction of ~40% reduction compared to littermates, were used for all experiments, as previously described in detail [26,38,39,40]. These mice were a generous gift from Professor Jerry Lingrel in the Department of Molecular Genetics, Biochemistry and Microbiology, at the University of Cincinnati College of Medicine. Mice were infused with either vehicle or TCB (4 weeks at 0.1 ug/g/day) intraperitoneally via osmotic minipumps (Alzet^®^ model 1004) [25]. All studies were approved by the Cleveland Clinic Institutional Animal Care and Use Committee.

### 2.2. Assessment of Blood Pressure and Renal Function

Conscious blood pressure was monitored at the indicated times using the tail-cuff method (IITC Life Science, Woodland Hills, CA, USA) [19]. Mice were placed in metabolic cages for collection of 24-h urine prior to euthanasia and collection of organs and plasma for histologic and biochemical analysis of renal structure and function. For metabolic cage studies, mice were allowed to acclimate to the cage for 48 h prior to urine collection. Blood samples were collected via cardiac puncture. Plasma cystatin C concentrations were determined by ELISA (R&D Systems, Minneapolis, MN, USA). Twenty-four-hour urine protein was determined using a Bradford protein assay (Biorad, Hercules, CA, USA). Urinary excretion of TCB was performed using a competitive fluoroimmunoassay (dissociation enhanced fluoroimmunoassay (DELFIA)), as previously described in detail [20,41,42]. The TCB DELFIA assay was based on utilizing a TCB-glycoside-thyroglobulin conjugate and TCB antiserum (anti-TCB, 1:10,000) obtained from rabbits immunized with TCB–bovine serum albumin (BSA) conjugate and secondary (goat anti-rabbit) europium labeled antibody (Perkin-Elmer, Waltham, MA, USA).

### 2.3. Reagents, Cell Culture, and Quantitative Polymerase Chain Reaction (PCR)

TCB (>98% by HPLC) was obtained from Herbest Bio-Tech (Baoji City, Shannxi, China). The human HK-2 cell line, as well as murine SYF fibroblasts in which Src family kinases are knocked-out (SYF) and those in which Src has been reconstituted (SYF + Src), was obtained from American Tissue Type Culture Collection (ATCC, Manassas, VA, USA) and maintained in culture according to the manufacture’s protocol. Primary normal human renal mesangial cells (NHMCs) were obtained from Lonza (Allendale, NJ, USA), used between passage four and eight and maintained in culture, according to the manufacture’s protocol, using culture media and reagents from Lonza. The NHMCs were modified fibroblast-like smooth muscle cells between capillaries isolated in the renal glomerulus and characterized by the manufacturer by positive immunofluorescence stain for fibronectin and negative for the epithelial markers cytokeratin 18 and 19 and negative for the endothelial marker von Willebrand (Factor VIII) antigen. Quantitative real-time PCR was used to measure gene expression using both TaqMan^®^ probes (Life Technologies, Carlsbad, CA, USA) with 18S rRNA used as the internal control and RT2 Profiler PCR Arrays (Qiagen, Germantown, MD, USA) according to manufacturers’ protocol. Then, PCR reactions were prepared as follows using all Qiagen reagents: 2x RT2 SYBR Green ROX FAST Mastermix (1150 μL), cDNA synthesis reaction (102 μL), and RNase-free water (1048 μL). Twenty microliters of the PCR reaction mixture were loaded into each well of the RT2 Profiler PCR array and arrays were carefully sealed using a Qiagen Rotor-Disc Heat Sealer and Rotor-Disc Heat-Sealing Film. After the run was completed, the cycle threshold (CT) was calculated using the Qiagen real-time cycler software. CT values were exported to an Excel spread sheet and analysis was performed using Qiagen web-based software.

### 2.4. Quantitative Histology

Mason’s trichome and picosirius red staining was performed on deparafinized 5-µm serial kidney sections. Quantitative morphometric analysis was performed on fields (at least 8 from each animal), and the collagen volume was determined using automated and customized algorithms/scripts for batch analysis (ImageIQ Inc., Cleveland, OH, USA) written for Image Pro Plus 7.0, as we have described in detail [25]. Trichrome analysis was also confirmed using the Total Collagen Assay Kit (Perchlorate-Free) from Biovision (San Francisco, CA, USA) and measurements were assessed according to manufacturer’s protocol.

### 2.5. Patient Study

In order to determine circulating TCB levels in a relevant clinical population, we measured TCB levels in 21 patients (≥18 years) seen at the Cleveland Clinic with a clinical diagnosis of CKD (CKD Stage 3 to 5, eGFR range 8–53 mL/min/1.73m^2^). The study protocol to process de-identified plasma samples obtained from standard clinical follow-up was approved by the Cleveland Clinic Institutional Review Board. Associated clinical data and standard of care laboratory values were collected from the patient’s medical records into a de-identified, Institutional Review Board-approved biospecimen registry. In a separate protocol, thirteen apparently healthy volunteer participants (mean age 43 ± 12 years, 64% male, 15% black) without a history of CKD served as non-CKD controls. They were prospectively recruited outside of any healthcare institution setting and did not report any active medical conditions at the time of blood draw. The study protocol was approved by the Cleveland Clinic Institutional Review Board, and written informed consent was obtained from each of the study participants before their participation in the study.

### 2.6. Biochemical Assays

Blood samples were collected in lithium-heparin plasma vacutainers at the time of clinical evaluation and were aliquotted and stored at −80 °C until analysis. Plasma samples were extracted for TCB measurements using C18 SepPak cartridges (Waters Inc., Cambridge, MA, USA) and TCB levels were measured using the DELFIA assay, as previously described in detail [20,41,42]. 

### 2.7. Statistical Analysis 

Normally distributed continuous variables were summarized as mean ± standard error of the mean if normally distributed. Data were first tested for normality using the D’Agostino-Pearson omnibus test. For data which did not pass the normality test, the Tukey test (for multiple groups) or the Mann–Whitney Rank Sum test was used. If the data passed the normality test, parametric comparisons were performed. If more than two groups were compared, one-way analysis of variance was performed prior to comparison of individual groups with the unpaired Student’s *t*-test with Bonferroni’s correction for multiple comparisons. If only two groups of normal data were compared, the Student’s *t*-test was used without correction. Statistical analysis was performed using GraphPad Prism^®^ (La Jolla, CA, USA) and statistical significance was considered as *p* < 0.05.

## 3. Results

### 3.1. Telocinobufagin-Induced Renal Fibrosis and Dysfunction Depends on Na^+^/K^+^-ATPase Signaling In Vivo 

Infusion of TCB yielded approximately 10-fold elevations in TCB from basal levels in both WT (110 ± 8 pmol/24 h) and NKA α-1^+/−^ mice (109 ± 9 pmol/24 h) and generated comparable levels to that seen by feeding mice a high-salt diet (4% NaCl) for 4 weeks (approximately 110 pmol/24 h). Similarly, TCB infusion for 4 weeks resulted in a significant increase from controls in systolic blood pressure in both WT (92 ± 3 vs. 158 ± 3 mmHg, *p <* 0.01) and NKA α-1^+/−^ mice (96 ± 3 vs. 159 ± 7 mmHg, *p <* 0.01).

**Figure 1:**
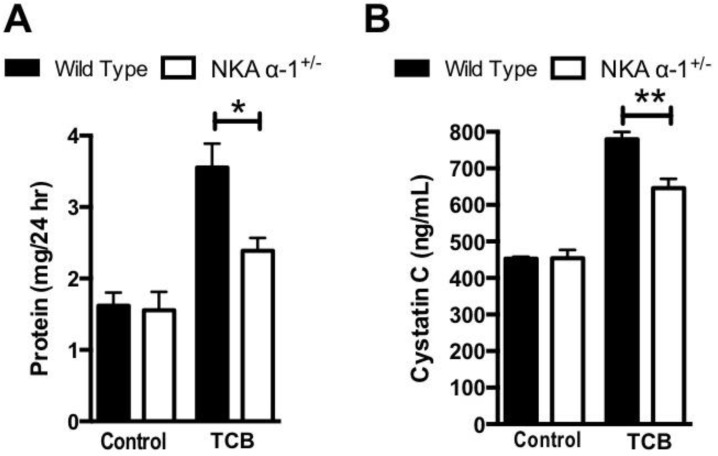
Na^+^/K^+^-ATPase alpha-1 (NKA α-1) knockdown attenuates Telocinobufagin (TCB)-induced renal dysfunction. Twenty-four-hour urine protein (**A**) and plasma cystatin C (**B**) in wild-type and NKA α-1^+/−^ mice treated with vehicle or TCB for 4 weeks; *n* = 4–8 mice/group, * *p <* 0.05, ** *p <* 0.01.

We next measured twenty-four-hour urine protein excretion after 4-week TCB infusion. The NKA α-1^+/−^ mice excreted significantly less urinary protein at 4 weeks compared to the WT controls (Figure 1A). Similarly, the NKA α-1^+/−^ mice had lower plasma cystatin C levels at 4 weeks compared to the WT controls (Figure 1B).

**Figure 2:**
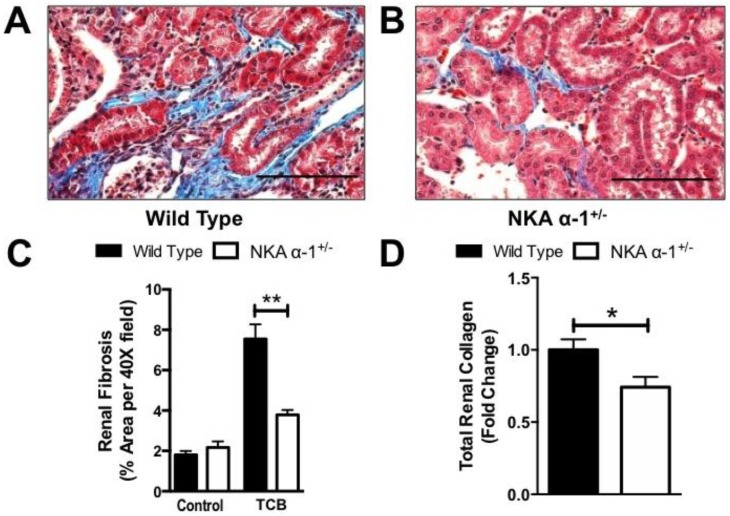
NKA α-1 knockdown attenuates TCB-induced increases in renal fibrosis. Representative Mason’s trichrome histology (**A**,**B**) and quantification (**C**,**D**) from wild-type and NKA α-1^+/−^ mouse kidneys after TCB infusion for 4 weeks. Scale bars are 50 micrometers, * *p <* 0.05, ** *p <* 0.01.

**Figure 3:**
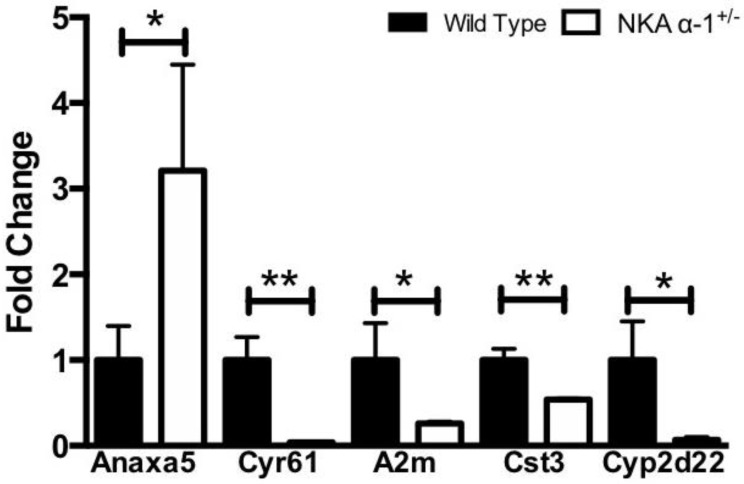
NKA α-1 knockdown alters TCB-induced changes in key genes associated with renal injury. Quantitative PCR from wild-type and NKA α-1^+/−^ mouse kidneys after TCB infusion for 4 weeks for markers of renal injury including apoptosis (*Anxa5*, annexin A5), extracellular matrix (*CYR61*, cysteine-rich protein 61), nephrotoxicity (*A2m*, alpha-2-macroglobulin), tissue remodeling (*Cst3*, cystatin C), and xenobiotic metabolism (*Cyp2d22*, cytochrome P450, family 2, subfamily d, polypeptide 22). Arrays were run with kidney cDNA from *n* = 2–3 pooled samples per array and *n* = 3 arrays per group. * *p <* 0.05, ** *p <* 0.01.

Next, kidneys of mice infused with TCB were sectioned and trichrome stained in order to examine them histologically for evidence of renal injury. Here, we noted that TCB infusion resulted in mild to moderate periglomerular and peritubular fibrosis in the renal cortex (Figure 2A,B), similar to what we observed after 4 weeks of marinobufagenin infusion in the rat [24]. NKA α-1^+/−^ mice had less renal fibrosis at 4 weeks compared to the WT controls as assessed by both quantitative morphometry (Figure 2C) and biochemical determination of total collagen content of kidney homogenate (Figure 2D). In order to further assess renal injury in this model, we performed a quantitative real-time PCR array on kidneys from both wild-type and NKA α-1^+/−^ mice infused with TCB. Here, we noted significant alterations between wild-type and NKA α-1^+/−^ kidneys in key genes related to apoptosis (annexin A5), extracellular matrix (cysteine-rich protein 61), nephrotoxicity (Alpha-2-macroglobulin), tissue remodeling (Cystatin C), and xenobiotic metabolism (cytochrome P450, family 2, subfamily d, polypeptide 22) (Figure 3). The complete comparison of renal injury genes assessed is presented (see Supplementary Materials, Supplemental Table S1). 

### 3.2. Pro-Fibrotic Effects of Telocinobufagin Depend on Na^+^/K^+^-ATPase Signaling

**Figure 4:**
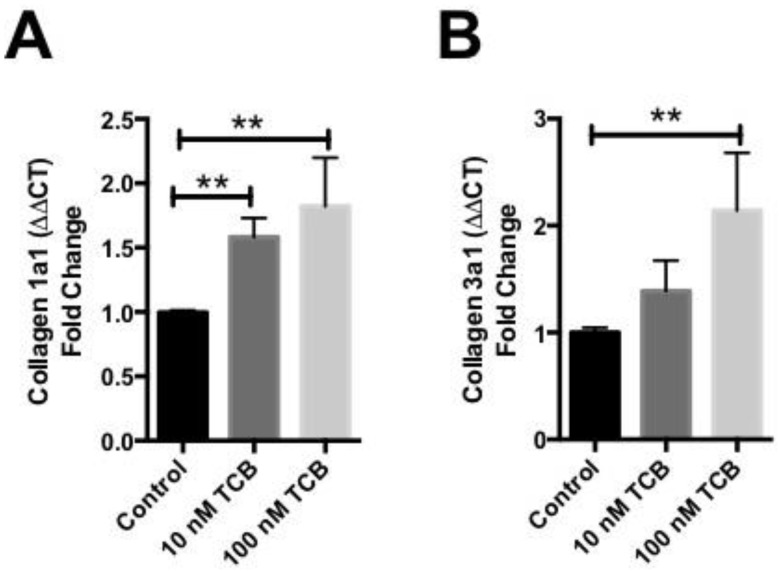
TCB induces increases collagen expression in human renal HK2 cells. Collagen 1 (**A**) and Collagen 3 (**B**) mRNA in HK2 proximal tubular cells after 24-h treatment with TCB. Data were summarized from *n* ≥ 3 experiments per group, * *p <* 0.05, ** *p <* 0.01.

**Figure 5:**
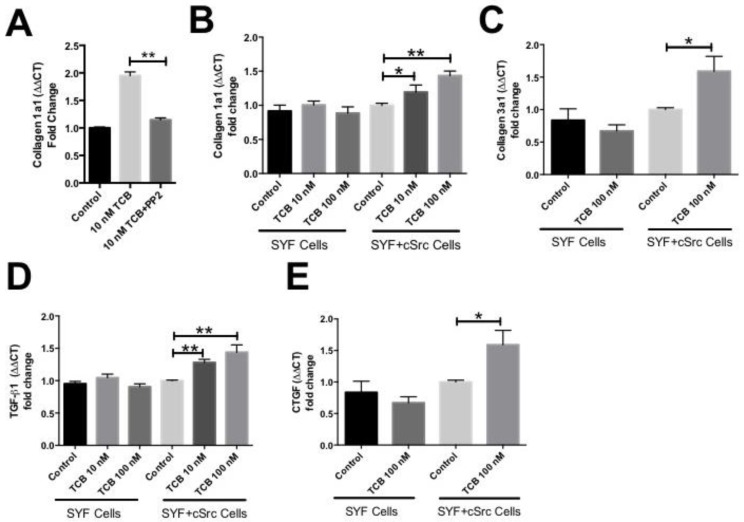
TCB-induced pro-fibrotic effects involve Src kinase. TCB-induced increases of Collagen 1 in normal human mesangial cells is attenuated by pretreatment with Src kinase inhibitor PP2 (30 min, 1 μM) (**A**). TCB-induced increases of Collagen 1 (**B**), TGFβ (**C**), Collagen 3 (**D**), and CTGF (**E**) mRNA in murine SYF + Src fibroblasts is attenuated in the absence of Src kinase (SYF cells). Data summarized from *n* ≥ 3 experiments per group, * *p <* 0.05, ** *p <* 0.01.

In in vitro experiments using the HK2 human renal proximal tubular cell line, 24-h treatment of cells with TCB (10–100 nM) produced dose-dependent increases in Collagen 1 (Figure 4A) and Collagen 3 (Figure 4B) expression as measured by quantitative real-time PCR. Additionally, we used primary NHMCs to examine TCB effects on collagen production in a renal fibroblast-like cell type. Twenty-four-hour treatment of NHMCs with TCB (10 nM) produced increases in Collagen 1 (Figure 5A) in this cell type as well. 

We next sought to determine the involvement of Src kinases in TCB-induced collagen production using several biochemical and genetic approaches. In NHMC’s pretreatment with the Src kinase, inhibitor PP2 (1 µM, 30 min) attenuated the TCB-induced increase in Collagen 1 at 24 h (Figure 5A). Next, we treated SYF fibroblasts in which Src family kinases were knocked-out (SYF) and those in which cSrc has been reconstituted (SYF + Src). Treatment of SYF fibroblasts with 100 nM TCB for 24 h did not induce significant increases in Collagen 1 (Figure 5B) or Collagen 3 (Figure 5C), as well as common pro-fibrotic growth factors such as transforming growth factor beta (TGFβ, Figure 5D), and connective tissue growth factor (CTGF, Figure 5E). Alternatively, in SYF + Src fibroblasts, TCB treatment resulted in significant increases in Collagen 1 (Figure 5B), Collagen 3 (Figure 5C), TGFβ (Figure 5D), and CTGF (Figure 5E). 

### 3.3. Telocinobufagin is Elevated in Human Chronic Kidney Disease

**Figure 6:**
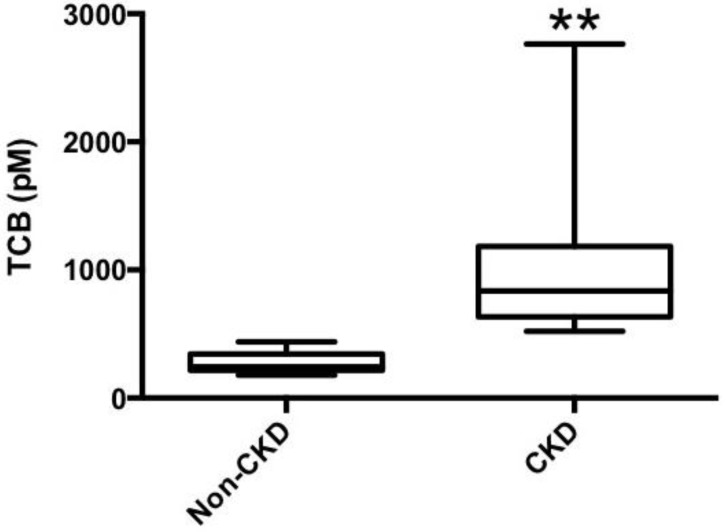
Comparison of plasma Telocinobufagin (TCB) levels between non-Chronic Kidney Disease (CKD) participants and CKD participants, ** *p* < 0.01.

Table 1 illustrates the baseline characteristics of the CKD patients examined in this study. Plasma TCB levels were elevated in patients with CKD compared to non-CKD controls (Figure 6). In our study cohort, there was no significant association between TCB and creatinine (*p* = 0.519) or the estimated glomerular filtration rate (*p* = 0.071).

## 4. Discussion

The current study provided several lines of evidence demonstrating that the pro-fibrotic renal effects of a novel, potent cardiotonic steroid, TCB, are mediated by signaling through the Na^+^/K^+^-ATPase. We showed in a mouse infusion model that, reduction of the receptor for CTS, the Na^+^/K^+^-ATPase, in the heterozygous NKA α-1^+/−^ mice, was able to attenuate renal collagen accumulation, proteinuria and decreased renal function of long-term administration of TCB, despite similar increases in systolic blood pressure to the wild-type mice. We also provided evidence in two renal cell types that TCB is able to directly stimulate a pro-fibrotic phenotype. Furthermore, using a combination of genetic and biochemical approaches, we showed that the signaling events leading to increased collagen accumulation depended on activation or Src kinase, a hallmark of Na^+^/K^+^-ATPase signaling. Finally, in our human patient study, TCB levels were elevated in CKD vs non-CKD patients. Taken together with our previous findings, these observations suggested that the elevated CTS levels, which accompany edematous states like CKD, may promote downstream adverse renal consequences. Thus, modulation of CTS levels or activity may present a novel therapeutic target in this population of patients, which is burdened with significant renal dysfunction.

### 4.1. Mechanisms Linking Cardiotonic Steroids to Renal Dysfunction

While the exact pathways which accelerate CKD have not been fully elucidated, it is believed that pro-inflammatory and pro-fibrotic mechanisms dominate in this setting [43,44]. To be sure, the Na^+^/K^+^-ATPase is a key signaling molecule involved in a number of inflammatory conditions in both human and animal models [19,45,46,47,48]. The association of CTS with inflammatory markers is not without precedent. Cao and coworkers demonstrated that TCB significantly enhances natural killer cell and peritoneal macrophage activation including induction of several interleukins, interferon-gamma and tumor necrosis factor-alpha [49]. We have similarly reported that the CTS ouabain induces increases in inflammatory cytokine expression from both macrophage and renal proximal tubular cell types [19]. Ouabain has also been shown to regulate pro-inflammatory cytokine expression at both the transcriptional [50,51] and post-transcriptional level [52,53]. Furthermore, Berendes and coworkers reported that elevations in endogenous ouabain are associated with increased concentrations of serum creatinine, as well as increases in several pro-inflammatory mediators, including tumor necrosis factor-alpha, interleukin-1beta, interleukin-2, interleukin-6, C-reactive protein and serum amyloid A in a study of over 400 critically ill patients. In this study, hospital mortality rates were also 64 times higher in patients with elevated endogenous ouabain levels [54]. Our group has also demonstrated that elevated levels of MBG, independent of established clinical risk factors, are associated with cardiac dysfunction, oxidative/nitrative stress, and worse event-free survival in patients with heart failure [25]. Additionally, heart failure patients who experience decreases in MBG levels over the course of hospitalization have significantly better event-free survival than those who have increases in MBG, and infusion of MBG in a mouse model recapitulates the increased oxidative/nitrative stress, cardiac fibrosis, and dysfunction seen in the human study [25]. Such findings suggest new avenues for understanding the pathophysiological role for CTS in pro-inflammatory settings. 

CTS have been implicated as important mediators of inflammation and fibrosis in animal models of cardiorenal disease as well. In partial (5/6th) nephrectomy, increases in circulating levels of MBG stimulate cardiac fibrosis in both rat and mouse [16,20,25,26,27,55]. Rats subjected to 5/6th nephrectomy develop systemic oxidant stress that is similar to that observed in rats subjected to MBG infusion, as evidenced by significant elevation of both plasma and left ventricular carbonylated protein. In rodent models, active and passive immunization against MBG [20,56], reduction of circulating levels of MBG by adrenalectomy [16], or pharmacologic interruption of the CTS mediated signaling [26,27,57,58] substantially attenuates both 5/6th nephrectomy and MBG infusion-mediated cardiac fibrosis and oxidant stress, an effect that is independent of blood pressure [55]. Further, CTS, such as MBG and ouabain, have been shown to stimulate [^3^H] proline incorporation, as well as gene and protein expression of collagen in several cell types, including primary cultured rat cardiac and human dermal fibroblasts as well as rat renal fibroblasts [17]. In fibroblasts, CTS-induced Na^+^/K^+^-ATPase signaling and oxidative stress are necessary for collagen production, which is effectively blocked not only by ROS scavenging and Src inhibition [27], but also through possible competitive inhibition of CTS binding to Na^+^/K^+^-ATPase by spironolactone and canrenone [16,20,59,60] or inhibition of an mTOR mediated pathway with rapamycin [57]. Thus, the pro-inflammatory and pro-fibrotic CTS-Na^+^/K^+^-ATPase signaling axis may provide a novel therapeutic target in settings, such as CKD where elevated CTS may induce renal inflammation and fibrosis.

### 4.2. Study Limitations

It is important to note that the in vivo experiments carried out in these studies were obtained from mice backcrossed onto the Black Swiss genetic background, which may account for some of the renal phenotypic differences noted between this strain and other studies using inbred C57/BL6 mice. For instance, the fact that the Black-Swiss strain carries two renin genes while the C57/BL6 only carries one has been suggested as a key difference, which distinguishes renal and cardiovascular phenotypes between these strains [61,62] including susceptibility to renal fibrosis [63]. This may explain in part the fibrotic and proteinuric phenotype noted in the Black Swiss strain. Furthermore, while the patient study was matched for sex, the lack of controlling for important confounding variables, such as age and race, may contribute to the differences observed between the non-CKD and CKD cohorts.

## 5. Conclusions

The CTS TCB has been demonstrated to be able to induce renal collagen accumulation and renal dysfunction in a Na^+^/K^+^-ATPase dependent manner as well as activating a pro-inflammatory and pro-fibrotic Na^+^/K^+^-ATPase/Src kinase signaling cascade in renal cell types. Furthermore, plasma levels of TCB were elevated in patients with CKD. Taken together with our understanding of the hypertrophic, oxidant, and fibrotic mechanisms induced by CTS, the current study suggests TCB may be an important diagnostic and therapeutic target in CKD progression, which may present opportunities to decrease the significant burden of renal dysfunction and mortality in these patients.

## Figures and Tables

**Table 1:** Baseline patient characteristics of Chronic Kidney Disease (CKD) patients (*n* = 21).

Variable	Value
**Demographics:**	
Age (years)	72 ± 11
Male gender, *n* (%)	11 (52%)
Black, *n* (%)	16 (76%)
**Laboratory data:**	
TCB (pM)	1001 ± 110
Creatinine (mg/dL)	4.1 ± 2.2
Sodium (mEq/L)	140 ± 3
eGFR (mL/min/1.73 m^2^)	25 ± 14

Values are mean ± standard deviation; Abbreviations: TCB, telocinobufagin; eGFR, estimated glomerular filtration rate.

## Supplementary Materials

Supplementary materials can be found at http://www.mdpi.com/1422-0067/19/9/2566/s1. Supplemental Table S1: Qiagen RT2 Profiler™ Mouse Nephrotoxicity PCR array comparing renal gene expression from TCB treated wild type and NKA α-1^+/−^ mice, Supplemental Figure S1: Structural comparison of (A) Telocinobufagin and (B) Marinobufagenin.
