# Supplementary material for: Telocinobufagin, a Novel Cardiotonic Steroid, Promotes Renal Fibrosis via Na+/K+-ATPase Profibrotic Signaling Pathways
Source: Int J Mol Sci. 2018 Aug 29;19(9):2566. doi: 10.3390/ijms19092566 (PMC6164831; doi:10.3390/ijms19092566)
Supplement: Supplementary file 1 [file ijms-19-02566-s001.pdf]

International Journal of *Molecular Sciences*

MDPI

Supplementary Material

Telocinobufagin, a Novel Cardiotonic Steroid, Promotes Renal Fibrosis via Na^+/K^+-ATPase Profibrotic Signaling Pathways

David J. Kennedy [1], Fatimah K. Khalaf [1], Brendan Sheehy [2], Malory E. Weber [2], Brendan Agatisa-Boyle [2], Julijana Conic [2], Kayla Hauser [2], Charles M. Medert [2], Kristen Westfall [2], Philip Bucur [2], Olga Fedorova [3], Alexei Y. Bagrov [4], W. H. Wilson Tang [2,5,6,*]

[1] Department of Medicine, University of Toledo College of Medicine, Toledo, Ohio
[2] Department of Cellular and Molecular Medicine, Lerner Research Institute Cleveland Clinic, Cleveland, Ohio
[3] Laboratory of Cardiovascular Science, National Institute on Aging, National Institutes of Health, Baltimore, Maryland
[4] Sechenov Institute of Evolutionary Physiology and Biochemistry, St. Petersburg, Russia
[5] Center for Cardiovascular Diagnostics and Prevention, Lerner Research Institute Cleveland Clinic, Cleveland, Ohio
[6] Department of Cardiovascular Medicine, Heart and Vascular Institute, Cleveland Clinic, Cleveland, Ohio
* Correspondence: 9500 Euclid Avenue, J3-4, Cleveland, OH 44195; E-mail: tangw@ccf.org; Tel.: +216-444-2121; Fax: +216-445-6165

Received: 9 August 2018; Accepted: 27 August 2018; Published: 29 August 2018

Table S1. Qiagen RT² Profiler™ Mouse Nephrotoxicity PCR array comparing renal gene expression from TCB treated wild type and NKA α-1$^{+/-}$ mice.

Gene	Wild Type (Relative Gene Expression, Mean ± SD)	NKA α-1$^{+/-}$ (Relative Gene Expression, Mean ± SD)	p value
**Apoptosis**			
Anxa5	1 ± 0.4	3.21 ± 1.24	**0.042**
Ghr	1 ± 0.19	0.54 ± 0.17	0.059
Igfbp3	1 ± 0.45	0.29 ± 0.29	0.078
Rtn4	1 ± 1.02	0.51 ± 0.02	0.443
Bmp4	1 ± 0.91	0.38 ± 0.31	0.318
Cd44	1 ± 1.02	0.83 ± 0.37	0.787
Bmp1	1 ± 0.36	1.03 ± 0.06	0.909
Cdkn1a	1 ± 1.03	1.17 ± 0.44	0.812
Btg2	1 ± 0.69	1.84 ± 0.58	0.184
Cd24a	1 ± 0.53	0.27 ± 0.19	0.085
Angptl4	1 ± 1	1.19 ± 0.3	0.769
Clu	1 ± 1.15	0.65 ± 0.15	0.622
Nqo1	1 ± 1.72	0.04 ± 0.03	0.383
Cat	1 ± 1.45	0.11 ± 0.05	0.346
**Cell cycle**			
Ccng1	1 ± 1.41	0.19 ± 0.17	0.379
Ccnd1	1 ± 1.07	0.35 ± 0.08	0.353
Egf	1 ± 1.48	1.8 ± 1.78	0.584
**Cell Proliferation**			
Nox4	1 ± 0.76	0.54 ± 0.46	0.418
Uchl1	1 ± 0.17	0.79 ± 0.15	0.172
Vcam1	1 ± 0.34	0.71 ± 0.1	0.223
Timp1	1 ± 0.86	0.6 ± 0.03	0.461
**Cytoskeleton Regulators**			

Sprr1a	1 ± 1.26	1 ± 0.6	0.997
Tmsb10	1 ± 0.62	0.89 ± 0.01	0.768
**Extracellular matrix**			
Lgals3	1 ± 0.86	0.03 ± 0.03	0.118
Mgp	1 ± 0.21	1.09 ± 0.21	0.647
Cyr61	1 ± 0.27	0.04 ± 0.01	0.**004**
Mt1	1 ± 0.19	0.75 ± 0.11	0.103
Ccl3	1 ± 0.46	0.27 ± 0.11	0.051
**Genes related to nephrotoxicity**			
A2m	1 ± 0.43	0.26 ± 0.02	**0.039**
Aass	1 ± 1.57	0.15 ± 0.14	0.399
Aldh1a1	1 ± 1.68	0.02 ± 0.01	0.367
Calb1	1 ± 1.35	0.02 ± 0.01	0.273
G6pc	1 ± 0.71	0.43 ± 0.01	0.221
Ipmk	1 ± 1	0.14 ± 0.01	0.210
Klk1	1 ± 0.96	0.11 ± 0.01	0.181
Ugt1a1	1 ± 1.12	1.43 ± 0.16	0.552
**Metal ion binding**			
Ccs	1 ± 0.63	0.78 ± 0.24	0.598
Ctss	1 ± 0.45	0.7 ± 0.13	0.323
Rgn	1 ± 1.69	0.54 ± 0.54	0.674
Scd1	1 ± 0.66	0.63 ± 0.21	0.403
**Oxidative stress**			
Gatm	1 ± 1.74	0.01 ± 0.01	0.374
G6pdx	1 ± 1.34	0.09 ± 0.09	0.304
**Tissue remodeling**			
Fn1	1 ± 1.01	0.61 ± 0.61	0.590
Igfbp1	1 ± 0.45	4.56 ± 3.54	0.160
Cst3	1 ± 0.13	0.54 ± 0.01	**0.003**
**Transporters**			
Abcb1a	1 ± 0.09	0.78 ± 0.42	0.414
Abcc2	1 ± 0.47	0.71 ± 0.17	0.364
Slc22a1	1 ± 0.67	0.73 ± 0.25	0.539
Slc22a5	1 ± 1.2	0.27 ± 0.25	0.355
**Xenobiotic metabolism**			
Cyp2d22	1 ± 0.45	0.07 ± 0.03	**0.022**
Fmo2	1 ± 1.26	0.14 ± 0.02	0.299

Arrays were run with kidney cDNA from n = 2–3 pooled samples per array and n = 3 arrays per group. Reference sequence number, Gene abbreviation, and Gene description for genes included in the Qiagen RT² Profiler™ PCR Array for Mouse Nephrotoxicity are listed below:

NM_175628 A2m	Alpha-2-macroglobulin
NM_013930 Aass	Aminoadipate-semialdehyde synthase
NM_011076 Abcb1a	ATP-binding cassette, sub-family B (MDR/TAP), member 1A
NM_013806 Abcc2	ATP-binding cassette, sub-family C (CFTR/MRP), member 2
NM_013467 Aldh1a1	Aldehyde dehydrogenase family 1, subfamily A1
NM_020581 Angptl4	Angiopoietin-like 4
NM_009673 Anxa5	Annexin A5
NM_007498 Atf3	Activating transcription factor 3
NM_016668 Bhmt	Betaine-homocysteine methyltransferase
NM_009755 Bmp1	Bone morphogenetic protein 1
NM_007554 Bmp4	Bone morphogenetic protein 4
NM_007570 Btg2	B-cell translocation gene 2, anti-proliferative
NM_009788 Calb1	Calbindin 1
NM_009804 Cat	Catalase
NM_011337 Ccl3	Chemokine (C-C motif) ligand 3
NM_007631 Ccnd1	Cyclin D1

NM_009831 Ccng1	Cyclin G1
NM_016892 Ccs	Copper chaperone for superoxide dismutase
NM_009846 Cd24a	CD24a antigen
NM_009851 Cd44	CD44 antigen
NM_007669 Cdkn1a	Cyclin-dependent kinase inhibitor 1A (P21)
NM_013492 Clu	Clusterin
NM_007752 Cp	Ceruloplasmin
NM_009976 Cst3	Cystatin C
NM_021281 Ctss	Cathepsin S
NM_021274 Cxcl10	Chemokine (C-X-C motif) ligand 10
NM_203320 Cxcl3	Chemokine (C-X-C motif) ligand 3
NM_206537 Cyp2c54	Cytochrome P450, family 2, subfamily c, polypeptide 54
NM_019823 Cyp2d22	Cytochrome P450, family 2, subfamily d, polypeptide 22
NM_010516 Cyr61	Cysteine rich protein 61
NM_010113 Egf	Epidermal growth factor
NM_181849 Fgb	Fibrinogen beta chain
NM_018881 Fmo2	Flavin containing monooxygenase 2
NM_010233 Fn1	Fibronectin 1
NM_008061 G6pc	Glucose-6-phosphatase, catalytic
NM_008062 G6pdx	Glucose-6-phosphate dehydrogenase X-linked
NM_007836 Gadd45a	Growth arrest and DNA-damage-inducible 45 alpha
NM_010255 Gamt	Guanidinoacetate methyltransferase
NM_025961 Gatm	Glycine amidinotransferase (L-arginine:glycine amidinotransferase)
NM_008096 Gc	Group specific component
NM_010284 Ghr	Growth hormone receptor
NM_008131 Glul	Glutamate-ammonia ligase (glutamine synthetase)
NM_053110 Gpnmb	Glycoprotein (transmembrane) nmb
NM_027127 Gpx8	Glutathione peroxidase 8 (putative)
NM_029555 Gstk1	Glutathione S-transferase kappa 1
NM_013541 Gstp1	Glutathione S-transferase, pi 1
NM_134248 Havcr1	Hepatitis A virus cellular receptor 1
NM_010442 Hmox1	Heme oxygenase (decycling) 1
NM_010443 Hmox2	Heme oxygenase (decycling) 2
NM_010480 Hsp90aa1	Heat shock protein 90, alpha (cytosolic), class A member 1
NM_010497 Idh1	Isocitrate dehydrogenase 1 (NADP+), soluble
NM_008341 Igfbp1	Insulin-like growth factor binding protein 1
NM_008343 Igfbp3	Insulin-like growth factor binding protein 3
NM_027184 Ipmk	Inositol polyphosphate multikinase
NM_010639 Klk1	Kallikrein 1
NM_008491 Lcn2	Lipocalin 2
NM_010705 Lgals3	Lectin, galactose binding, soluble 3
NM_008567 Mcm6	Minichromosome maintenance deficient 6 (MIS5 homolog, S. pombe) (S. cerevisiae)
NM_008597 Mgp	Matrix Gla protein
NM_013602 Mt1	Metallothionein 1
NM_001285833 Nox4	NADPH oxidase 4
NM_130456 Nphs2	Nephrosis 2 homolog, podocin (human)
NM_008706 Nqo1	NAD(P)H dehydrogenase, quinone 1
NM_016978 Oat	Ornithine aminotransferase
NM_013614 Odc1	Ornithine decarboxylase, structural 1
NM_009060 Rgn	Regucalcin
NM_194053 Rtn4	Reticulon 4
NM_009127 Scd1	Stearoyl-Coenzyme A desaturase 1
NM_009202 Slc22a1	Solute carrier family 22 (organic cation transporter), member 1
NM_011396 Slc22a5	Solute carrier family 22 (organic cation transporter), member 5
NM_008766 Slc22a6	Solute carrier family 22 (organic anion transporter), member 6
NM_007707 Socs3	Suppressor of cytokine signaling 3
NM_013671 Sod2	Superoxide dismutase 2, mitochondrial
NM_011435 Sod3	Superoxide dismutase 3, extracellular

NM_009263 Spp1	Secreted phosphoprotein 1
NM_009264 Sprr1a	Small proline-rich protein 1A
NM_011593 Timp1	Tissue inhibitor of metalloproteinase 1
NM_025284 Tmsb10	Thymosin, beta 10
NM_013749 Tnfrsf12a	Tumor necrosis factor receptor superfamily, member 12a
NM_011670 Uchl1	Ubiquitin carboxy-terminal hydrolase L1
NM_201645 Ugt1a1	UDP glucuronosyltransferase 1 family, polypeptide A1
NM_145079 Ugt1a6a	UDP glucuronosyltransferase 1 family, polypeptide A6A
NM_011693 Vcam1	Vascular cell adhesion molecule 1
NM_011701 Vim	Vimentin
NM_007393 Actb	Actin, beta
NM_009735 B2m	Beta-2 microglobulin
NM_008084 Gapdh	Glyceraldehyde-3-phosphate dehydrogenase
NM_010368 Gusb	Glucuronidase, beta
NM_008302 Hsp90ab1	Heat shock protein 90 alpha (cytosolic), class B member

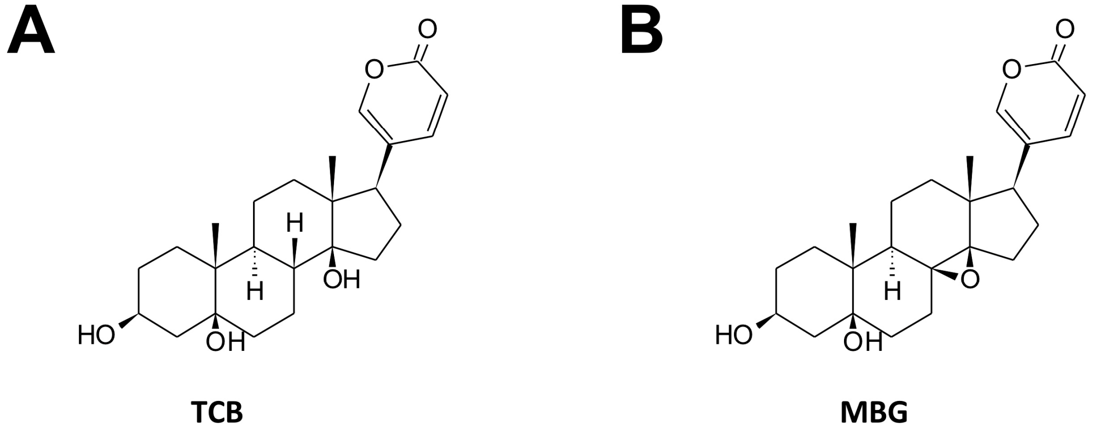

Figure S1. Structural comparison of (A) Telocinobufagin and (B) Marinobufagenin.
